# Eugenol Exerts Apoptotic Effect and Modulates the Sensitivity of HeLa Cells to Cisplatin and Radiation

**DOI:** 10.3390/molecules24213979

**Published:** 2019-11-03

**Authors:** Moustafa Fathy, Michael Atef Fawzy, Henning Hintzsche, Toshio Nikaido, Thomas Dandekar, Eman M. Othman

**Affiliations:** 1 Department of Regenerative Medicine, Graduate School of Medicine and Pharmaceutical Sciences, University of Toyama, Toyama 930-0194, Japan; moustafa_fathyy@yahoo.com (M.F.); tnikaido@med.u-toyama.ac.jp (T.N.); 2 Department of Biochemistry, Faculty of Pharmacy, Minia University, 61519 Minia, Egypt; michaelfawzy777@yahoo.com; 3 Bavarian Health and Food Safety Authority, Eggenreuther Weg 43, 91058 Erlangen, Germany; hintzsche@toxi.uni-wuerzburg.de; 4 Institute of Pharmacology and Toxicology, University of Wurzburg, Versbacher Str. 9, 97078 Wurzburg, Germany; 5 Department of Bioinformatics, Biocenter, University of Würzburg, Am Hubland, 97074 Wuerzburg, Germany; dandekar@biozentrum.uni-wuerzburg.de

**Keywords:** eugenol, HeLa cells, cisplatin, radiation, apoptosis

## Abstract

Eugenol is a phytochemical present in different plant products, e.g., clove oil. Traditionally, it is used against a number of different disorders and it was suggested to have anticancer activity. In this study, the activity of eugenol was evaluated in a human cervical cancer (HeLa) cell line and cell proliferation was examined after treatment with various concentrations of eugenol and different treatment durations. Cytotoxicity was tested using lactate dehydrogenase (LDH) enzyme leakage. In order to assess eugenol’s potential to act synergistically with chemotherapy and radiotherapy, cell survival was calculated after eugenol treatment in combination with cisplatin and X-rays. To elucidate its mechanism of action, caspase-3 activity was analyzed and the expression of various genes and proteins was checked by RT-PCR and western blot analyses. Eugenol clearly decreased the proliferation rate and increased LDH release in a concentration- and time-dependent manner. It showed synergistic effects with cisplatin and X-rays. Eugenol increased caspase-3 activity and the expression of *Bax*, cytochrome c (Cyt-c), caspase-3, and caspase-9 and decreased the expression of *B-cell lymphoma (Bcl)-2*, cyclooxygenase-2 (*Cox-2*), and interleukin-1 beta (IL-1β) indicating that eugenol mainly induced cell death by apoptosis. In conclusion, eugenol showed antiproliferative and cytotoxic effects via apoptosis and also synergism with cisplatin and ionizing radiation in the human cervical cancer cell line.

## 1. Introduction

Current cancer treatments are very efficient drugs, yet these medications result in a number of serious side effects [1,2]. In the search for novel therapies, one promising approach is the use of natural compounds that stop, delay, or reverse the initiation and progression of carcinogenesis. Some of these agents act synergistically with established cytostatic drugs or radiation and thus can be considered chemo- and/or radio-sensitizers [3,4]. Many previous studies have shown the in vivo and in vitro cytostatic activity of cloves [5,6]. Eugenol, 4-allyl-2-methoxyphenol, is an aromatic phytochemical present as the primary constituent of clove oil (*Syzygium aromaticum*, Myrtaceae) [7]. In addition to its use as a food additive and scent in makeup [8], it has been generally utilized as a part of conventional medications in numerous Asian nations as an antiseptic, pain relieving, or antibacterial agent [9]. In the United States, clove oil has been advertised as a dental pain-relieving agent and germicide, and is used in mouthwashes and pharmaceutical drugs, and furthermore as a fixing agent in fragrance-based agents [10]. Different investigations have shown antiviral effects of eugenol [11] and antiproliferative effects of eugenol-related compounds [12]. Eugenol showed antioxidant activity at low concentrations; however, at high concentrations, it enhanced the generation of free radicals acting as a pro-oxidant, leading to cell death [13]. It was reported that eugenol affected the metabolic profile of normal human oral cells in vitro and caused cellular damage for hepatocytes isolated from rats [14,15]. Eugenol at high concentrations was also observed to increase the DNA breaks in normal human fibroblast cells [16]. Studies of eugenol showed its different effects on cancer cells; firstly, cancer prevention action by its antioxidant effect and secondly, pro-oxidant action that kills cancer cells by affecting several signaling pathways [17]. Eugenol regulates many molecular targets to mediate its cytotoxic effect as it inhibits nuclear factor kappa-light-chain-enhancer of activated B cells (NF-κB) activation, downregulates prostaglandin synthesis, decreases cyclooxygenase-2 (*Cox-2*) activity, produces cell cycle arrest in the S phase, increases the generation of reactive oxygen species (ROS), diminishes *B-cell lymphoma (Bcl)-2* level, and reduces inflammatory cytokine levels, resulting in apoptotic cell death [13,18,19,20,21,22]. Investigations of the impacts of eugenol on different cancer cell lines showed apoptosis induction in human melanoma G361 cells mediated by increased expression of caspase-3 and caspase-6. Apoptosis in mast cells was triggered by translocation of p53 to the mitochondria [23]. For human leukemia cells (HL60), a decrease in the mitochondrial membrane potential following the activation of caspase-3 triggered apoptosis [24]. In human colon cancer HT-29 cells, eugenol increased ROS, while decreasing thiol levels [25]. Furthermore, eugenol inhibited growth and cyclooxygenase-2 expression, showing an antiproliferative effect on colon cancer cells (HCT-15 and HT-29) [20]. In a dose-dependent manner, eugenol also induced apoptosis of human breast cancer MCF-7 cells, accompanied by exhaustion in glutathione (GSH) and an increase in ROS [26].

The second most common malignancy in women is cervical cancer [27]. Despite the fact that radiotherapy is a compelling treatment methodology, the pelvis is the most widely recognized site of failure [28,29,30]. Surgery, chemotherapy and radiotherapy are well-known options for cervical cancer treatment [31,32]. Although FDA has endorsed the use of two chemotherapeutic agents (topotecan and cisplatin) for cervical cancer end stages, serious problems including neutropenia, anemia, and thrombocytopenia frequently occur [22]. Considering the antiproliferative activity of eugenol, its anticancer activity and molecular mechanism were evaluated in a cervical cancer cell line hoping for the development of a promising strategy by its combination with cisplatin or radiotherapy for cancer treatment. The present study investigates the molecular mechanism of eugenol underlying its antiproliferative effects on human cervical cancer cells (HeLa cells) and their increase in sensitivity to common therapies.

## 2. Results

### 2.1. Eugenol Cytotoxicity on HeLa Cells

#### 2.1.1. Effect of Eugenol on Survival of HeLa Cells

**Figure 1:**
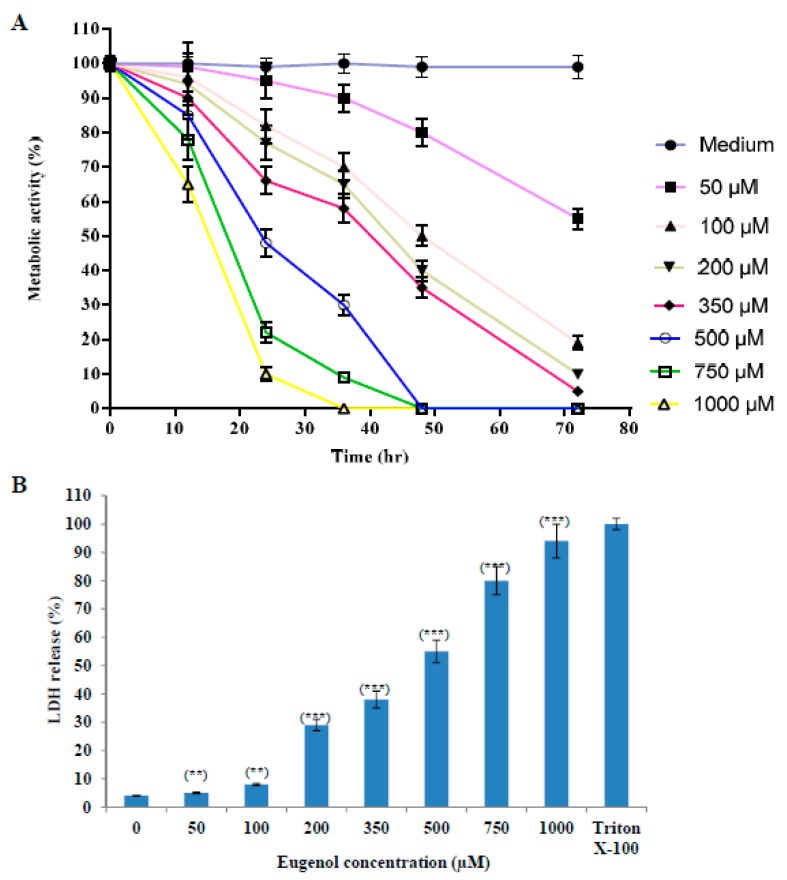
Eugenol cytotoxicity effect on HeLa cells. (**A**) Effect of eugenol on the proliferation of HeLa cells is concentration- and time-dependent. Proliferation of HeLa cells treated, in triplicate, with different concentrations (50, 100, 200, 350, 500, 750, and 1000 µM) of eugenol for different time intervals was estimated by CCK-8 and expressed as a percentage relative to that of cells cultured in medium only. Data represent mean ± standard deviation (SD). CCK-8, Cell Counting Kit-8. (**B**) Eugenol increases LDH enzyme leakage in HeLa cells in a concentration-dependent manner. HeLa cells were treated, in triplicate, with different concentrations (0–1000 µM) of eugenol for 24 h. LDH release (cytotoxicity) was expressed as a percent relative to that of 10% Triton X-100-treated cells. Data represent mean ± SD. Significant difference was analyzed by one-way ANOVA test, where ** *p* < 0.01 and *** *p* < 0.001 compared to HeLa cells treated with 0 µM eugenol. LDH, lactate dehydrogenase.

The proliferation of cells was reduced after treatment with increasing concentrations (50–1000 µM) of eugenol as shown in Figure 1A. The proliferation was expressed as a percentage relative to that of cells cultured in medium only. We used 350 μM concentration of eugenol for all our subsequent experiments because it produced the optimal effect of about 66 ± 4% survival after 24 h treatment.

#### 2.1.2. Effect of Eugenol on Lactate Dehydrogenase (LDH) Release from HeLa Cells

The effect of different concentrations (50–1000 µM) of eugenol on LDH release from HeLa cells for 24 h (cytotoxicity) is shown in Figure 1B. LDH release is expressed as a percent relative to that of Triton X-100-treated cells. Our results showed that treatment of HeLa cells with increasing concentrations of eugenol induced a significant release of LDH from HeLa cells in a dose-dependent manner of 38 ± 3% and 94 ± 6% at 350 and 1000 µM, respectively, indicating disruption of the cell membrane structure. Eugenol at 350 µM was used for all the subsequent experiments.

### 2.2. Effect of Eugenol on Sensitivity of HeLa Cells to Cisplatin

**Figure 2:**
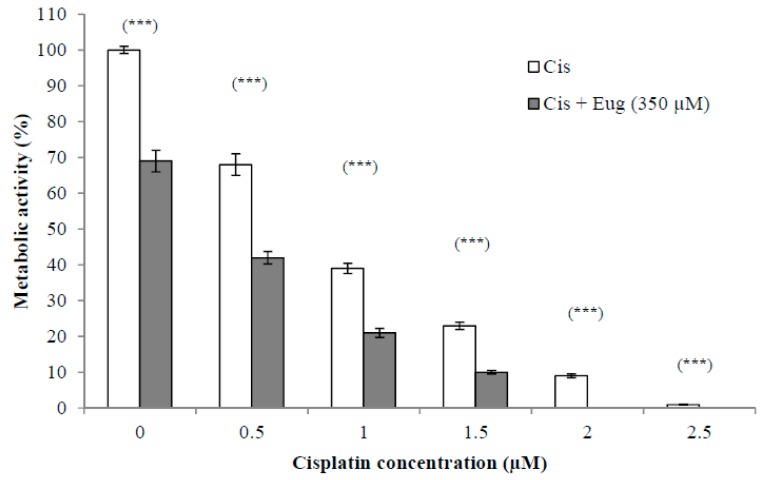
Effect of eugenol on sensitivity of HeLa cells to cisplatin. HeLa cells were treated, in triplicate, with different concentrations (0–2.5 µM) of cisplatin alone or in combination with eugenol (350 µM) for 24 h. The survival of cells, estimated by CCK-8, was expressed as a percentage relative to that of cells treated with medium only (without cisplatin or eugenol). Data represent mean ± SD. Significant difference was analyzed by one-way ANOVA test, where *** *p* < 0.001 compared to cells not treated with eugenol.

The use of established anticancer agents in combination with natural products may show increased antitumor activity due to sensitization of the cancer cells to the anticancer agents induced by the effect of these natural products. This could lead to fewer side effects due to using lower doses. The effect of different concentrations (0.5–2.5 µM) of cisplatin alone or in combination with eugenol (350 µM) on HeLa cell viability for 24 h is shown in Figure 2. The survival of cells was expressed as a percentage relative to that of cells treated with medium only (without cisplatin or eugenol). The results indicated that the combination of eugenol and cisplatin showed a significant (*p* < 0.001) decrease in cell viability at all given concentrations. Eugenol increased significantly the sensitivity of HeLa cells to cisplatin at all cisplatin concentrations when compared to cells treated with cisplatin alone. These results support the idea that the combination of these drugs has a higher efficacy than monotherapy with either cisplatin or eugenol.

### 2.3. Effect of Eugenol on Sensitivity of HeLa Cells to radiation

**Figure 3:**
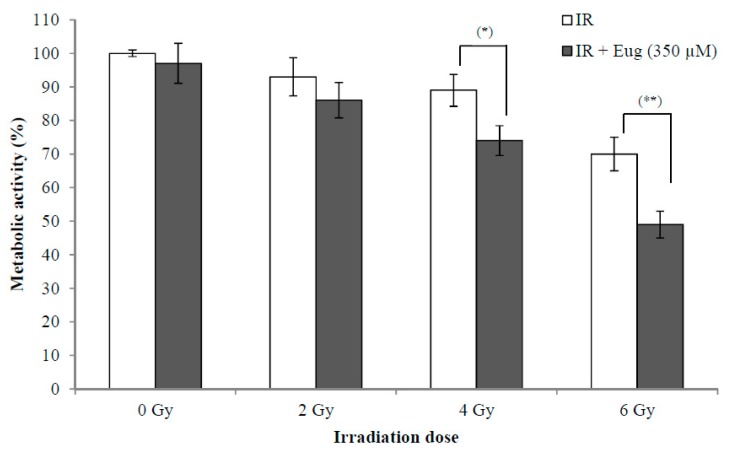
Effect of eugenol on sensitivity of HeLa cells to radiation. HeLa cells were treated, in triplicate, with eugenol (350 µM) for 6 h and then irradiated with (0, 2, 4, and 6 Gy) X-rays and the medium was replaced with a fresh medium. The survival of cells after 24 h, estimated by CCK-8, was expressed as a percentage relative to that of non-radiated cells treated with medium only. Data represent mean ± SD. * *p* < 0.05 and ** *p* < 0.01 compared to cells not treated with eugenol.

The use of radiotherapy for cancer treatment has many adverse effects on adjacent normal cells. The synergistic effect between radiation and natural products such as eugenol is interesting because this might lead to the use of lower radiation doses resulting in fewer side effects. The effect of eugenol treatment (350 µM) for 6 h and then irradiation with (0, 2, 4, and 6 Gy) X-rays on HeLa cell proliferation is shown in Figure 3. The survival of cells after 24 h is expressed as a percentage relative to that of non-irradiated cells treated with medium only. The results showed that eugenol significantly increased the sensitivity of HeLa cells to irradiation at 4 Gy (*p* < 0.05) and 6 Gy (*p* < 0.01), while it failed to increase the sensitivity of HeLa cells at 2 Gy irradiation. The survival of cells irradiated with only 2, 4, and 6 Gy was 93.1 ± 5.6%, 89.3 ± 4.7% and 70.2 ± 5.1%, respectively, while irradiation (with 2, 4, and 6 Gy) after eugenol treatment decreased the survival of cells to 86.4 ± 5.2%, 74.5 ± 4.4%, and 49.3 ± 4.3%, respectively. These results showed that the proliferation inhibition ability of the combination of eugenol and radiation could potentially allow the reduction of radiotherapy doses and side effects.

### 2.4. Effect of Eugenol on Cell Signaling of HeLa Cells

#### 2.4.1. Effect of Eugenol on Caspase-3 Activity in HeLa Cells

**Figure 4:**
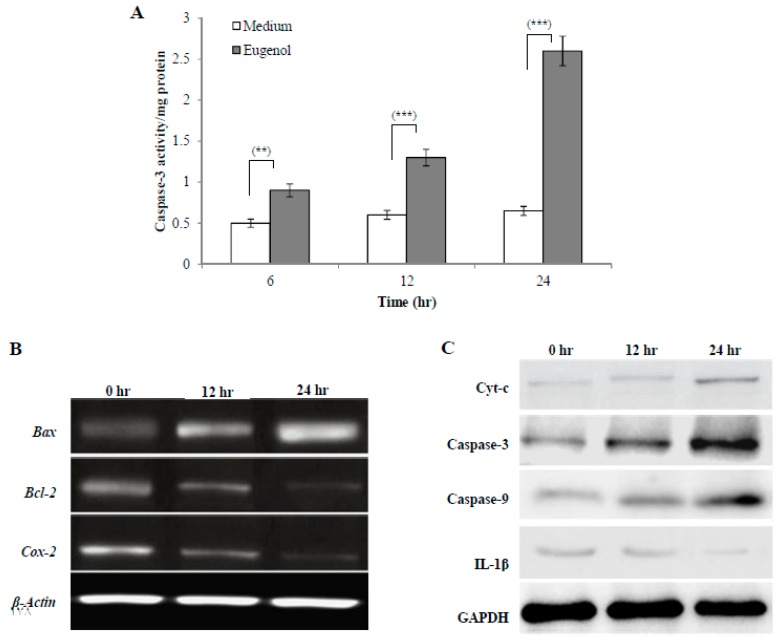
Effect of eugenol on cell signaling of HeLa cells. (**A**) Eugenol increases caspase-3 activity in HeLa cells. HeLa cells were treated with medium or eugenol (350 µM)-containing medium for 6, 12, or 24 h. Activity was detected by a caspase-3 colorimetric activity assay kit. Data, obtained from three independent experiments, represent mean ± SD. Significant difference was analyzed by one-way ANOVA test, where ** *p* < 0.01 and *** *p* < 0.001 compared to HeLa cells treated with medium only. (**B**) Effect of eugenol on *Bax*, *Bcl-2*, and *Cox-2* gene expression in HeLa cells. HeLa cells were treated with medium or eugenol (350 µM)-containing medium for 12 or 24 h. *β-actin* was used as the internal control. (**C**) Effect of eugenol on Cyt-c, caspase-3, caspase-9, and IL-1β protein expression in HeLa cells. Representative immunoprecipitation blots of protein expression of HeLa cells treated with medium or eugenol (350 µM)-containing medium for 12 or 24 h. GAPDH was used as the internal control.

Caspases are a family of proteases that trigger apoptosis. During the early stages of apoptosis, caspase-3 is activated and then it mediates the cleavage and activation of many key proteins involved in the apoptosis cascade. The effect of treatment with medium alone or eugenol (350 µM)-containing medium for 6, 12, or 24 h on caspase-3 activity is shown in Figure 4A. The results showed that eugenol significantly increased caspase-3 activity in HeLa cells lysates in a time-dependent manner when compared to cells treated with medium only.

#### 2.4.2. Effect of Eugenol on *Bax*, *Bcl-2*, and *Cox-2* Gene Expression in HeLa Cells

To analyze molecular targets of eugenol in HeLa cells, *Bax*, *Bcl-2*, and *Cox-2* gene expression was assessed after treating HeLa cells with medium or eugenol (350 µM)-containing medium for 12 or 24 h. *β-actin* was used as the internal control. To confirm apoptosis induction by eugenol in HeLa cells and explore the apoptotic pathway that eugenol activates, the levels of expression of different pro- and anti-apoptotic genes such as *Bax* and *Bcl-2* were assessed. As shown in Figure 4B, untreated HeLa cells showed a relatively high expression of *Bcl-2* which decreased after treatment with eugenol for 12 and 24 h. The expression of *Bax*, which is a pro-apoptotic gene, was increased following eugenol treatment. From the results, a time-dependent increase in the *Bax*/*Bcl-2* ratio after 12 and 24 h of eugenol treatment was noticed, suggesting that eugenol activates apoptosis through the mitochondrial pathway.

Additionally, the effect of eugenol treatment on the expression of *Cox-2* gene was investigated. The results showed that the expression of *Cox-2* gene was significantly decreased over time by eugenol treatment. This showed that eugenol inhibited the proliferation of HeLa cells.

Taken together, these results indicated that *Bcl-2*, *Bax*, and *Cox-2* gene expression was affected by eugenol.

#### 2.4.3. Effect of Eugenol on Cytochrome c (Cyt-c), Caspase-3, Caspase-9, and Interleukin-1 Beta (IL-1β) Protein Expression in HeLa Cells

Representative immunoblots of protein (Cyt-c, caspase-3, caspase-9, and IL-1β) expression in HeLa cells treated with medium or eugenol (350 µM)-containing medium for 12 or 24 h are shown in Figure 4C. Glyceraldehyde 3-phosphate dehydrogenase (GAPDH) was used as the internal control. The results showed that eugenol increased the expression of all apoptotic proteins (Cyt-c, caspase-3, and caspase-9) in a time-dependent manner, suggesting eugenol-dependent induction of apoptosis via the mitochondrial pathway. Eugenol treatment significantly downregulated the (IL-1β) protein level in a time-dependent manner to nearly undetectable levels after 24 h.

## 3. Discussion

Many of the currently established cancer therapies are facing problems due to the many side effects as well as the development of resistance [33,34]. To overcome these problems, one approach is combination therapy with agents acting on different signaling pathways as this results in higher effectiveness, less side effects and toxicity, and higher survival rates [35,36]. Recent studies focused on dietary compounds such as genistein, eugenol, sulforaphane, curcumin, gallate, and resveratrol as promising agents due to their antiproliferative or cytotoxic activity [19,37,38,39,40]. When combined with these agents, traditional cancer therapies show a higher anticancer activity by synergism and so lower doses can be used, having the advantage of reducing systemic toxicity originated by chemotherapies or radiotherapies. Eugenol (4-allyl-2-methoxyphenol) is present in different spices such as bay, cinnamon, and clove leaves as a main component of their essential oils [19]. Recently, due to its antioxidant, anti-inflammatory, and anticancer abilities, eugenol has been examined for different biological activities [7,41,42]. Previously, we described the great safety of eugenol and the in vivo modulation of inducible nitric oxide synthase (iNOS) pathway by eugenol in carbon tetrachloride-induced liver injury and showed its downregulation effect on the expression of tumor necrosis factor (TNF)-α, interleukin-6 (IL-6), iNOS, and NF-κB [43]. Furthermore, authors are now investigating the effect of different concentrations of eugenol on the characteristics of adipose tissue-derived mesenchymal stem cells regarding their viability and migration abilities in vitro and the impact of this effect on their hepatic antifibrotic activity in vivo, obtaining highly promising results even at low concentrations of eugenol pre-treatment, but data have not been published yet.

In our study, eugenol treatment produced a significant dose-dependent and time-dependent antiproliferative activity against HeLa cells, confirmed by viability assays and LDH release, which is in accordance with other studies showing similar eugenol effects on different human cancer cells [18,20,44,45,46]. Moreover, we show here that eugenol significantly increases the sensitivity of HeLa cells to cisplatin at all cisplatin concentrations used in this study. Also, eugenol significantly increased the sensitivity of HeLa cells to radiation (4 and 6 Gy). These results of eugenol-induced chemo- and radio-sensitization were found in different cancer cell lines by many signaling pathways [47,48].

Our experiments provide a well-controlled baseline on which the following detailed analysis of the potential effects of eugenol were conducted, identifying caspase-3, caspase-9, *Bcl-2*, and cytochrome release as well as effects on mediators of inflammation (*Bax*, *Cox-2*, and IL-1β) as key targets of eugenol action.

Moreover, lowering the radiation or chemotherapy doses depends on a synergistic effect between these measures and eugenol. Co-administration of eugenol could minimize the adverse effects of chemotherapy or radiation by lowering the applied doses. More experiments are planned to check several combinations using Chou-Talalay method and study in detail the possible molecular mechanisms for such combinatorial effects of eugenol.

Apoptosis or programmed cell death is an ordered process with cascade activation of many proteins leading to cell death [49]. The inability to induce apoptosis may lead to the induction of mutations which may be followed by carcinogenesis. However, and more importantly, the observed cytotoxicity, indicated by LDH release which is induced by necrosis or late apoptosis, is an important aspect for finding therapeutic drugs to treat cancer [50,51]. In order to investigate the apoptotic pathway induced by eugenol toward HeLa cells, caspase-3 activity was assessed. Caspase-3 initiates programmed cell death by acting on different proteins which lead to apoptotic, morphological, and biological changes [52]. Our results show that eugenol significantly increases caspase-3 activity in HeLa cell lysates in a time-dependent manner. These results are in accordance with the results of previous studies on other cancer cells [52,53,54].

Eugenol triggered the cleavage of caspase-3, with a significant increase in its active forms confirming apoptosis induction. To confirm that eugenol triggers apoptosis through the mitochondrial pathway, we assessed the levels of caspase-9 that showed a significant increase with time in the caspase-9 active form. Together, these results show that eugenol induces apoptosis in HeLa cells through the internal mitochondrial pathway by the activation of caspase-9 and caspase-3.

*Bcl-2* is an anti-apoptotic gene and has been involved in many cancers such as melanoma, breast, lung, and liver carcinomas. It is also believed to have a role in cancer treatment resistance [55,56]. Hence, a search for promising cytotoxic agents capable of reducing *Bcl-2* gene expression is necessary. The present results of eugenol effects on *Bcl-2* gene expression in HeLa cells confirmed a significant reduction in gene expression. Therefore, eugenol could induce and promote the antitumor activity of common chemotherapeutic agents and radiation. *Cox-2* gene, which is involved in the production of prostaglandins, is known to have a role in inflammation and its high level has been associated with many cancers [57,58]. *Cox-2* mRNA expression was highly increased in many cancer cells and this overexpression was found to be one of the causes of apoptosis resistance [26,59], so *Cox-2* is considered as a target for chemopreventive agents. This study investigated the effect of eugenol on the expression of this cancer-related gene to explore its antiproliferative activity on HeLa cells. The expression of *Cox-2* gene in this study showed a significant decrease over time by eugenol treatment. This confirms that eugenol, in part, inhibits the proliferation of HeLa cells by targeting *Cox-2* gene in cancer treatment strategies.

IL-1β, a member of the interleukin-1 cytokine family, is a central inflammatory mediator. It has a role in many cellular processes such as immune response induction, prostaglandin synthesis and release, cellular division, and apoptosis. IL-1β became an important therapeutic target in different inflammatory diseases, immunity disorders, Alzheimer’s disease, and various tumors [60,61]. Accordingly, IL-1β protein level was analyzed in this study in HeLa cells which showed that eugenol treatment significantly downregulated IL-1β protein expression in a time-dependent manner. With regards to these results of the pro-inflammatory cytokine (IL-1β) protein expression, which has a role in cellular division, the antiproliferative effect of eugenol was, in part, attributed to its anti-inflammatory activity with downregulation of this cancer-related protein.

## 4. Materials and Methods

### 4.1. Chemicals, Reagents, and Antibodies

Dulbecco’s Modified Eagle’s Medium (DMEM), L-glutamine, phosphate-buffered saline (PBS, pH = 7.4), eugenol (>99% pure; molecular weight, 164.20), cisplatin, deoxyribonuclease I (DNase I), and Tween 20 were obtained from Sigma-Aldrich, Inc (St Louis, MO, USA). A protease inhibitor cocktail was purchased from Roche (Mannheim, Germany). Fetal bovine serum (FBS) was purchased from Biosolutions International (Melbourne, Australia), while penicillin-streptomycin mixture and PureLink™ RNA Mini Kit were obtained from Invitrogen (Grand Island, NY, USA). The cell counting kit-8 (CCK-8) was purchased from Dojindo Molecular Technologies (Dojindo Co., Kumamoto, Japan). A lactate dehydrogenase (LDH) cytotoxicity assay kit was obtained from Cayman Chemical (Ann Arbor, MI, USA) and a caspase-3 colorimetric activity assay kit was obtained from Millipore (Kankakee, IL, USA). The ReverTra Ace qPCR RT Kit was purchased from Toyobo Co., Ltd. (Osaka, Japan) and a Taq DNA polymerase kit was obtained from QIAGEN (Hilden, Germany). p-Nitroanilide (pNA) and the primary antibodies against Cyt-c, IL-1β, caspase-3, caspase-9, and glyceraldehyde 3-phosphate dehydrogenase (GAPDH) were obtained from Santa Cruz (CA, USA), while the secondary antibodies conjugated with horseradish peroxidase were purchased from Life Technologies Japan (Tokyo, Japan).

### 4.2. Cell Culture and Reagents

HeLa cells, obtained from the American Type Culture Collection (ATCC: CCL-2, Manassas, VA, USA), were maintained in fresh DMEM supplemented with 10% FBS, l-glutamine (10 g/L), and penicillin-streptomycin mixture (10 g/L). Cells were cultured at 37 °C in a 5% CO_2_ humidified incubator. Eugenol was kept at room temperature and diluted with the culture medium to the required concentrations immediately before use for the treatment of HeLa cells.

### 4.3. Cell Viability Assay

Cell counting kit-8 (CCK-8) was used to quantitatively evaluate cell proliferation. Briefly, the cells were seeded in triplicate at a density of 5 × 10^3^ cells per well in 96-well plates and allowed to grow in fresh DMEM medium for 24 h. The cells were then washed with PBS and the medium was changed to fresh DMEM containing different concentrations (0–1000 µM) of eugenol. After the specified time of incubation, the cells were washed again with PBS, then 100 µL of DMEM medium with 10% WST-8 (2-(2-methoxy-4-nitrophenyl)-3-(4-nitrophenyl)-5-(2,4-disulfophenyl)-2*H*-tetrazolium) solution was added to the wells, and the plate was incubated for 2 h. Then, the absorbance of the wells at 450 nm was measured using the HTS Multi-Mode Microplate Reader (BioTek Instruments, Winooski, VT, USA). The absorbance is proportional to the number of viable cells in the medium. Cell proliferation at different time points was expressed as a percentage relative to that of cells treated with medium without eugenol.

### 4.4. Cytotoxicity Assay

LDH enzyme leakage into the culture medium is a well-known indicator of cell membrane injury and cell cytotoxicity. It was measured using the LDH cytotoxicity assay kit. Briefly, cells (5 × 10^3^ cells per well) were seeded in triplicate in 96-well plates and incubated overnight in fresh DMEM medium before changing the medium to a fresh one containing different concentrations (0–1000 µM) of eugenol. After 24 h, 100 μL of medium from each well was carefully transferred to new plates and 100 μL of LDH reaction solution, prepared according to the manufacturer’s instructions, was added to each well. Plates were gently shaken at 37 °C for 30 min, then, absorbance was measured at 490 nm. LDH release (cytotoxicity) was expressed as a percent relative to that of 10% Triton X-100-treated cells.

### 4.5. Chemotherapy Sensitivity Assay

To check if eugenol affected the sensitivity of HeLa cells to cisplatin, cell survival was examined after treating the cells with cisplatin alone or in combination with eugenol. Cells were seeded in triplicate at a density of 5 × 10^3^ cells per well in 96-well plates and incubated overnight in fresh DMEM medium. Then medium was changed to a fresh one containing different concentrations (0–2.5 µM) of cisplatin alone or in combination with eugenol (350 µM). After 24 h, cell survival was determined by CCK-8 as described above and was expressed as a percentage relative to that of cells treated with medium only (without cisplatin or eugenol). 

### 4.6. Radiotherapy Sensitivity Assay

To examine if eugenol increased HeLa cell sensitivity to radiation, cells (5 × 10^3^ cells per well) were seeded in triplicate in 96-well plates and allowed to grow in fresh DMEM medium for 24 h. They were treated with eugenol (350 µM)-containing medium for 6 h. Then, cells were irradiated with (0, 2, 4, and 6 Gy) X-rays generated with an X-ray apparatus (Hitachi Medico Technology Co., Kashiwa, Japan) operating at 150 kV and 120 mA and the medium was replaced with fresh medium. After 24 h, cell survival was determined by CCK-8 as described before and was expressed as a percentage relative to that of non-radiated cells treated with medium only (without eugenol).

### 4.7. Caspase-3 Activity Analysis

The caspase-3 colorimetric activity assay kit was used for the detection of caspase-3 activity. Briefly, cells were seeded in 25 cm^2^ tissue culture flasks and incubated overnight before being treated with medium or eugenol (350 µM)-containing medium for 6, 12, or 24 h. Then, cell lysates were prepared using the lysis buffer for caspase-3 activity assay according to the manufacturer’s instructions. Lysates were transferred to 96-well plates. The concentration of *para*-nitro aniline (pNA) released from the substrate, which was added and incubated for 1 h at 37 °C, by caspase-3 was calculated from the absorbance measured at 405 nm and from a calibration curve prepared with known pNA concentrations. Data were obtained from three independent experiments.

### 4.8. Expression Analysis of Bax, Bcl-2, and Cox-2 Genes by Reverse Transcription-PCR

Total RNA was extracted from untreated and eugenol-treated cells using the PureLink™ RNA Mini Kit according to the manufacturer’s instructions. The amount of RNA extracted was measured spectrophotometrically on NanoDrop 1000 (Thermo Scientific, Waltham, MA, USA). Aliquots of RNA were treated with 0.1 U/µL DNase I at room temperature for 15 min to remove genomic DNA. cDNAs were synthesized using 0.5 µg DNase I-treated RNA by using the ReverTra Ace qPCR RT Kit according to the manufacturer’s instruction. The resulting cDNA was subjected to PCR using the Taq DNA polymerase kit. PCR was performed with initial denaturation for 70 s at 95 °C, followed by 30 amplification cycles (denaturing for 45 s at 93 °C, annealing for 45 s at the specific annealing temperature, and elongation for 40 s at 72 °C) with final extension for 100 s at 72 °C, in a 25 μL reaction mixture according to the manufacturer’s instruction using the thermal cycler (Applied Biosystems, Gene Amp PCR System 2700). Primer sequences used and annealing temperatures are mentioned in Table 1. The *β-actin* primer was used as the internal control. Amplified products were separated on 2% agarose gels containing 0.1 μg/mL ethidium bromide and visualized by LAS-3000 (Fujifilm, Tokyo, Japan) and Multi-Gauge v3.0 software (Fujifilm, Tokyo, Japan).

### 4.9. Western Blot Assay of Cyt-c, Caspase-3, Caspase-9, and IL-1β Protein Expression

Protein expression levels were assessed by sodium dodecyl sulfate-polyacrylamide gel electrophoresis (SDS-PAGE) and immunoblotting. HeLa cells were cultured in six-well plates and treated with medium or eugenol (350 µM)-containing medium for 12 or 24 h as described above. Cells were then collected, washed, and lysed with RIPA lysis buffer containing 150 mM NaCl, 50 mM Tris-Cl, pH 7.5, 0.1% SDS, 0.5% sodium deoxycholate, 2 mM phenylmethylsulfonyl fluoride, and 1% Nonidet P-40, supplemented with the protease inhibitor cocktail. Cell lysates containing 50 µg of protein were separated by SDS-PAGE (15% acrylamide) and transferred onto polyvinylidene fluoride membranes (Millipore) which were blocked with 5% skimmed milk for 30 min at room temperature and washed with PBS containing 0.3% Tween 20 and then incubated overnight at 4 °C with the primary antibodies (1:500 dilution in PBS) for Cyt-c, IL-1β, caspase-3, and caspase-9. Bound primary antibodies were detected by incubating the membranes for 1 h with the respective secondary antibodies conjugated with horseradish peroxidase. Immunoreactive proteins were detected using an enhanced chemiluminescence system (Life Technologies Japan) according to the manufacturer’s instructions and visualized using a luminescent image analyzer (LAS-4000, Fujifilm Co., Tokyo, Japan). Each immunoblotting was repeated three times. GAPDH (1:1000 dilution) was used as the internal control to ensure equal loading and blotting. The Bio-Rad Trans-Blot SD Cell apparatus (Bio-Rad, Hercules, CA) was used in electrophoresis and electroblotting.

### 4.10. Statistical Analysis

All results were expressed as mean ± standard deviation. Statistical comparison was conducted using Student’s *t*-test after one-way analysis of variance (ANOVA) using GraphPad Prism 5 statistical software (GraphPad, La Jolla, CA, USA) and Excel software (Microsoft, Redwood, WA, USA). The results were considered to be significant when the probability values (*p*) were less than 0.05.

## 5. Conclusions

From this study, it is concluded that *Bcl-2*, *Bax*, *Cox-2*, and IL-1β expressions in HeLa cells are affected by eugenol. Eugenol may target IL-1β, which then blocks the downstream targets *Bcl-2* and *Cox-2*. Authors currently examine, but this is not completed yet, the eugenol molecular effects on other normal and cancer cell lines and these ongoing studies confirm in their trends the effects reported here. Thus, this study investigated the molecular mechanism of eugenol underlying its anticancer effect on human cervical cancer HeLa cells and their sensitivity increase to common therapies, demonstrating the ability of eugenol as a promising anti-inflammatory, anticarcinogenic, cytotoxic, and chemo- and radio-sensitizing agent acting through various signaling pathways.

## Figures and Tables

**Table 1:** Primers used in reverse transcription-PCR.

Primer	Sequence of the Primer	Annealing Temperature	Size (bp)
***Bax***	Forward: 5′-GTTTCATCCAGGATCGAGCAG-3′	53 °C	487
	Reverse: 5′-CATCTTCTTCCAGATGGTGA-3′		
***Bcl-2***	Forward: 5′-CCTGTGGATGACTGAGTACC-3′	53 °C	127
	Reverse: 5′-GAGACAGCCAGGAGAAATCA-3′		
***Cox-2***	Forward: 5′-ATACCAAAACCGCATTGCCG-3′	57 °C	305
	Reverse: 5′-TCTAACTCCGCAGCCATTTC-3′		
***β-Actin***	Forward: 5′-CGGGACCTGACTGACTAC-3′	56 °C	252
	Reverse: 5′-GAAGGAAGGCTGGAAGAG-3′		
